# A rare case of papillary cystadenoma of epididymis presented with painless scrotal mass

**DOI:** 10.22088/cjim.12.0.388

**Published:** 2021

**Authors:** Seyyed Hosein Ghasemi Shektaie, Hamid Shafi, Ali Falahi, Fatemeh Mahmoudlou, Emadoddin Moudi

**Affiliations:** 1 Student Research Committee, Babol University of Medical Sciences, Babol, Iran; 2 Cancer Research Center, Health Research Institute, Babol University of Medical Sciences, Babol, Iran; 3 Clinical Research Development Center, Shahid Beheshti Hospital, Babol University of Medical Sciences, Babol, Iran.; 4 Department of Urology, School of Medicine, Babol University of Medical Sciences, Babol, Iran

**Keywords:** Myxosarcoma, Transthoracic Echocardiogram (TTE)

## Abstract

**Background:**

It is a rare cardiac malignant primary tumor that seems to derive from the same cellular line as myxomas, but the prognosis is very different. It is a rare cardiac malignant primary tumor that seems to derive from the same cellular line as myxomas, but the prognosis is very different. It is a rare cardiac malignant primary tumor that seems to derive from the same cellular line as myxomas, but the prognosis is very different. Cardiac myxosarcoma is a rare neoplasm that appears to rise from the same cellular source like myxoma. It is difficult to differentiate a myxoma tumor from a myxosarcoma tumor because of its appearance and pathology examination. Myxosercoma tumor requires surgery and chemoradiotherapy, but myxoma is treated only by surgery.

**Case Presentation:**

We describe a case of a 58-year-old patient with a left atrium myxosarcoma, presenting with congestive heart failure. Transthoracic echocardiogram (TTE) showed a large polypoid and mobile mass in the left atrium, the patient underwent cardiac surgery and the tumor was successfully extracted, and histopathological result revealed typical features of myxoma. 15 days after surgery, he underwent explorative laparatomy because of progressive GI bleeding. Laparatomy revealed extensive metastatic masses in abdomen and the pathology diagnoses was myxosaroma. Unfortunately, in spite of supportive care, the patient expired on postoperative day one.

**Conclusion:**

It is difficult to differentiate a myxoma tumor from a myxosarcoma tumor because of its appearance and pathology examination. Maybe magnetic resonance imaging can help us to achieve more data suggesting malignancy.

Papillary cystadenoma of the epididymis (PCE) is a benign epithelial tumor located in the head of the epididymis and formed by a proliferation of the epithelium inside ecstatic efferent ducts. However, it is the second most common benign tumor of epididymis after the adenomatous tumor, a rare epithelial tumor and occurs sporadically or in association with von Hippel-Lindau disease (VHL)(1-6). Most cases are incidental findings in patients with VHLD (7). Other cases are found in patients who seek consultation for infertility (oligozoospermia or obstructive azoospermia) or hydrocele (8). The most frequent symptom is a painless increase of the testicle. Forty percent of cases are bilateral and it is precisely these cases are most frequently associated with VHLD (9). The diagnosis can be made by ultrasound when observing a solid epididymal head with small cysts or echogenic foci associated (4). The prognosis is excellent (10). Only isolated cases have been reported in which a PCE metastasized in retroperitoneal lymph nodes as clear cell adenocarcinoma (11) in the absence of renal tumor or VHLD.

Up to 2018, there was no reporting of unilateral PCE as the initial presentation of VHLD (3). Patients with VHLD are at risk for developing multiple renal cysts and renal cell carcinomas (RCC), Retinal capillary hemangioblastomatosis (12), cerebellar hemangioblastoma (13). These clinical findings are actually considered sufficient to justify the diagnosis (14) however, still genetic testing is the standard method to diagnose VHL (15). The more important is about their initial presentations which most of them first came with  retinal hemangioblastomatosis followed by cerebellar hemangioblastoma (13). We described herein a rare case of PCE presented with painless scrotal mass. However, he did not accept to pass the genetic testing studies and also his IHC studies were negative about VHLD but had kind of a retinal hemangioma and a suspicious cerebellum mass.

## Case Presentation

A 37-year-old man has presented with a history of unilateral painless scrotal mass for many years. Also, he had a period of voiding difficulty and frequency. In the past medical history, he underwent a herniorrhaphy due to a right scrotal hernia in his sixth and right hydrocelectomy about 13 years ago. He has an 8-year-old daughter now and is not smoker. In his examination his kidneys were not palpable and no sign of VHLD detected. Lymphadenopathy was not palpated. There were diffused skin lipoma-like masses all over his body. The ultrasonography (US) of these masses about 2 years ago had shown multiple lipoma-like cyst in the left breast (about 10 to 30 mm in sizes around the nipple) and a benign left axillary lymph node (about 10 × 20mm in size).

The US of kidneys revealed multiple cysts in variable sizes in the cortex of the both kidneys (The biggest of them in the Lt. kidney was 39.5 × 30mm in size and in the right was about 21 × 18.5 mm). And also reported “it can be a sign of ADPKD and furthermore screening is needed”. The US of scrotum showed right testis shrinking with heterogeneous parenchyma (mild to moderate atrophy). Also in the head of the right epididymis, there was a hypervascular area (about 26 ×35mm in size and consists of multiple cysts separated by thick echogenic septa). Its report emphasized on “the adhesion of these cysts to the upper pole of the right testis Is presenting a neoplastic mass”. Besides, varicose veins were detected. 

The semen analysis result was totally in an infertile range. In details, it was liquefaction=more than 60min (normal range=less than 20 min), sperm count=less than 5 million/ml (the least for fertilization is more than 10 million per ml), viability=less than 35% (normal range=more than 55%). The tumor markers (LDH, alpha feto protein and beta HCG) were within normal range. The conventional laboratory data before the surgery were normal totally (FBS=100, BUN=13, CR=1.06, Na=141, K=4, WBC=6000, Hb=13.8).

Finally, the patient underwent right orchiectomy via inguinal approach because of the US’s report emphasizing on the adhesion of the multiple cysts of the right epididymis to the upper pole of the right testis which were presenting a neoplastic mass. Considering the pathological study, in the macroscopic view, the testis was creamy with a not-invaded capsule. It was about 4.2×3.5×3 cm in size. There were no signs of any suspicious changes in the spermatic cord. But in the supralateral of the epididymis, a 3 cm solid-cystic yellowish to brown tumoral mass was seen. The mass had lobulated borders with no obvious invasion to the testis.

**Figure 1:**
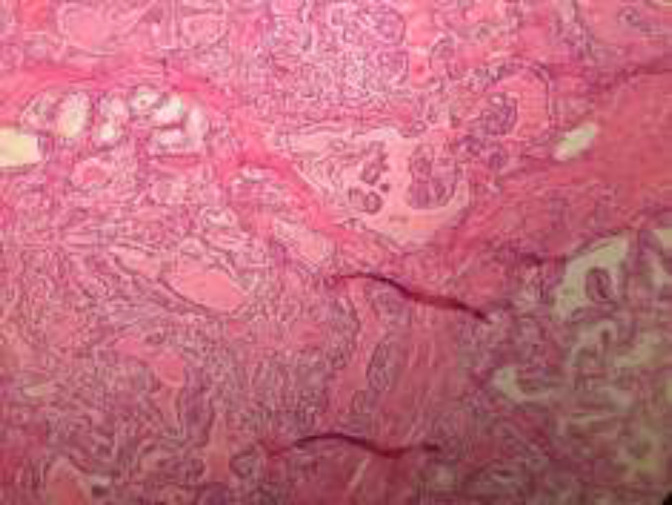
The tumor consists of a cystic space with papillary excrescences and hemorrhagic fluid (5× objective).

**Figure 2:**
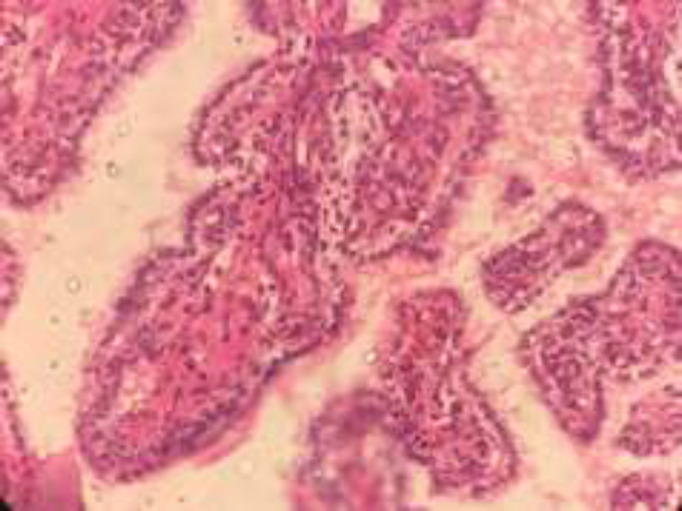
Tubular structures of cells with abundant clear cytoplasm

In the microscopic sections, cystic spaces containing colloid like eosinophilic material and papillary projections lined by a layer of epithelium (figure 1). Other areas showed sheets and tubular structures of cells with abundant clear cytoplasm (figure 2).

The pathology confirmed PCE and suggested for more studies for making it clear whether it is related to VHLD or not. Unfortunately, he did not accept to pass the genetic testing studies. But the IHC was negative for VHLD. The computed tomography (CT) of the abdominal and pelvic after the surgery was totally normal. Only there were multiple hypodense cystic foci in the cortex of the left kidney. The report mentions matching these cysts with the US’s results.

To date, 5 months after surgery, the patient is alive with no evidence of disease recurrence or metastasis signs. To consult the ophthalmologist, the suspicious dilatation in the retina could be an initial presentation of a retinal angioma. To consult the neurosurgeon, the brain MRI was a defined border mass in the para sagittal view of cerebellum without post contrast abnormal enhancement. However, the size of the mass was not noticeable but it could be an initial of a cerebellar hemangioblastoma. Altogether, the evidence shows a unilateral papillary cystadenoma of epididymis with an atrophied parenchymal of right testis, suggesting a clinical follow-up and more investigation for VHLD.

## Discussion

This was the first PCE in our center and the paramedic studies confirmed the clinical diagnosis and suggested for genetic and immunohistochemistry (IHC) studies. Unfortunately, our patient did not accept to pass the genetic testing but the IHC was negative for von Hippel-Lindau disease (VHLD). PCE is a benign neoplasm occurring mainly in young adult males (1) and originates from the efferent ductules of the head of the epididymis, however, it can arise from the tail of epididymis too (3, 4). Totally, the English-language reports of PCE since the first one made by Sherrick in 1956 (1) is about 70 cases till 2014 (4, 8). The age of the patients in the diagnosis time varies from 16 to 76 with a mean age of 35 (9). Mostly, PCE is unilateral and asymptomatic (9). Even the symptomatic patients present usually with a slow-growing painless scrotal swelling (4, 10) which is not tender in examination. But PCE can be presented by a painful nodule in the head of the epididymis too (9). 

Ultrasonography is the preferred modality for every testicular and epididymal mass (11-13) like PCE which is usually displayed as well circumscribed cystic nodule in the head of the epididymis that varies from 0.5 to 8 cm in size with an average of 2 cm (9). PCE can happen unilaterally or bilaterally. Bilateral PCE is pathognomonic of VHLD based on literature (3). But the relation between unilateral PCE and VHLD is still not clear. However unilateral PCE has been reported in the context of a known VHLD, has never been reported as the first presentation or in the context of an unknown VHLD (1, 2, 7). On the other hand, Miscia et al. (2018) reported a unilateral PCE as a first presentation of VHLD, nevertheless were not investigated for VHLD clues and only found out the main disease when the patient did a molecular analysis after he had been exposed for a brain computed tomography (CT) when there were some suspicious cerebellar lesions. Maybe if he was examined for VHLD at first they could find it out sooner though there were no guidelines yet. Maybe there is a necessity to introduce some risk factors such as age (5) or the initial clinical signs and symptoms like the presentations in eye, kidney and cerebellum (14) to find out the disease sooner and also reduces the life-threatening effects of VHLD in the patients. Still, we need more studies and following-up the patients to introduce some useful risk factors to determine which patient is more likely to have VHLD nonetheless,he presented with a PCE.
